# A systematic review of the international evolution of online mental health strategies and recommendations during the COVID-19 pandemic

**DOI:** 10.1186/s12888-022-04257-8

**Published:** 2022-09-20

**Authors:** Nerea Almeda, Diego Díaz-Milanés, Mencia R. Guiterrez-Colosia, Carlos R. García-Alonso

**Affiliations:** 1 Department of Psychology, Universidad Loyola Andalucía, Seville, Spain; 2 Department of Quantitative Methods, Universidad Loyola Andalucía, Cordova, Spain

**Keywords:** COVID-19, Strategies, Recommendations, Mental health, Systematic review, Longitudinal

## Abstract

**Introduction:**

The global health crisis caused by the COVID-19 pandemic has had a negative impact on mental health (MH). As a response to the pandemic, international agencies and governmental institutions provided an initial response to the population’s needs. As the pandemic evolved, the population circumstances changed, and some of these international agencies updated their strategies, recommendations, and guidelines for the populations. However, there is currently a lack of information on the attention given to response strategies by the different countries throughout the beginning of the pandemic.

**Objectives:**

1) To evaluate the evolution of online MH strategies and recommendations of selected countries to cope with the MH impact of COVID-19 from the early stages of the pandemic (15 April 2020) to the vaccination period (9 June 2021) and 2) to review and analyse the current structures of these online MH strategies and recommendations.

**Methodology:**

An adaptation of the PRISMA guidelines to review online documents was developed with a questionnaire for MH strategies and recommendations assessment. The search was conducted on Google, including documents from April 2020 to June 2021. Basic statistics and Student’s t test were used to assess the evolution of the documents, while a two-step cluster analysis was performed to assess the organisation and characteristics of the most recent documents.

**Results:**

Statistically significant differences were found both in the number of symptoms and mental disorders and MH strategies and recommendations included in the initial documents and the updated versions generated after vaccines became available. The most recent versions are more complete in all cases. Regarding the forty-six total documents included in the review, the cluster analysis showed a broad distribution from wide-spectrum documents to documents focusing on a specific topic.

**Conclusions:**

Selected governments and related institutions have worked actively on updating their MH online documents, highlighting actions related to bereavement, telehealth and domestic violence. The study supports the use of the adaptation, including the tailor-made questionnaire, of the PRISMA protocol as a potential standard to conduct longitudinal assessments of online documents used to support MH strategies and recommendations.

**Supplementary Information:**

The online version contains supplementary material available at 10.1186/s12888-022-04257-8.

## Background

Most countries in the world have been significantly affected by the COVID-19 outbreak, reporting a high number of infections and deaths since the pandemic started in March 2020 [1]. Therefore, this global health crisis has also had a negative impact on the mental health (MH) of the population [2]. According to the World Health Organisation (WHO), there is an international agreement on considering MH care and psychosocial support as key components of the designed COVID-19 response plan [3]. Despite the great efforts that are being made worldwide to provide guidance and advice, COVID-19 is having a major and negative impact on MH [4–6]. Increased uncertainties, worry, stress and perceived threat undermine the MH of the population [7]. Staying at home, social distancing and containment measures have significantly affected psychological stability and wellbeing [8], resulting in the following MH problems: acute stress disorder, depression, low mood, irritability, insomnia, sadness and posttraumatic stress symptoms, anger, anxiety, grief and/or confusion [9–11]. The results of a longitudinal evaluation in Spain reported an increase in anxiety, depression and stress levels during the lockdown of the first wave [12, 13]. In Italy, anxiety levels tripled, and depression also increased [14]. A recent systematic review showed that anxiety, depression, posttraumatic stress disorder, psychological distress and stress were prevalent during the pandemic in China, Spain, Italy, Iran, the United States of America, Turkey, Nepal and Denmark [15]. In the United States of America, during June 2020, the adult population manifested MH symptoms such as anxiety and depression (31%), started or increased substance use (13%), presented trauma or stressor-related disorder symptoms (26%) and suicidal thoughts (11%) [16]. The MH impact of COVID-19 also affects daily life activities, including nutrition and sexual activity [17]. Furthermore, the findings of a meta-analysis revealed that during pandemics, a large number of health care staff experience elevated levels of anxiety, depression, and insomnia [18].

Currently, the pandemic is evolving, and effective vaccines have been developed to address the physical health crisis [19–21]; however, the MH crisis remains a collective global task.

Addressing the MH impact of the COVID-19 crisis is a priority for the main international organisations. The WHO recommended the inclusion of MH and psychosocial issues in national COVID-19 responses [22]. To do so, governments should communicate reliable COVID-19 information while promoting psychosocial interventions and MH care [23, 24]. Considering this framework, it is also a priority to ensure the availability of emergency MH services, strengthen social cohesion, reduce isolation, promote psychological support, and protect the human rights of people suffering from severe MH disorders and psychosocial disabilities [22]. According to the WHO, a crucial way to recover is building affordable community-based services, including the coverage of MH services by insurance companies as well as investing in community care [22, 25]. In addition, research was highlighted as a key issue in the recovery process, as the analysis of MH care in this context is crucial [22].

The United Nations (UN) developed tips for supporting people suffering from mental disorders [26]. The organisation also included telecommuting tips and information on how to speak to children, access professional health care and access external MH care.

Last, the American Psychological Association (APA) approached the MH impact of COVID-19 by developing different strategies and posting them on their website [27], including how to prevent burnout in health care workers, enhance the development of a psychologically healthy work environment while promoting health among employees, and build confidence in vaccines by engaging the community. The APA also highlighted the key role of psychiatrists and psychologists in assisting people suffering from the physical and MH consequences of COVID-19 as well as supporting families with loved ones in an intensive care unit. In addition, the relevance of self-care to help parents communicate with teenagers and promote the use of telehealth is also emphasised.

However, there has been a lack of information on the evolution of online policies, strategies and guidelines designed by governments and international institutions (published in different formats such as web pages, reports, and documents) throughout the pandemic in response to the evolution of population needs [28, 29].

This research aims to 1) evaluate the evolution of online MH strategies and recommendations of selected countries to cope with the MH impact of COVID-19 from the early stages of the pandemic (15 April 2020) to the vaccination period (9 June 2021) and 2) review and analyse the current structures of these online MH strategies and recommendations. Online MH strategies and recommendations, the study target, are published in documents located in specific websites linked to governmental institutions.

## Methods

This section is divided into the following parts: a) methodology for document selection (search strategy and eligibility criteria), b) instrument, c) variable grouping, d) data collection procedure and, finally, e) data analysis.

### Methodology for document selection

#### Search strategy and eligibility criteria

The Preferred Reporting Items for Systematic Reviews and Meta-Analyses (PRISMA) guideline was used to carry out the systematic review [30]. Due to the urgent nature and the unpredictability of the COVID-19 situation, some adaptations were made. Although the PRISMA guideline was developed to evaluate the effects of interventions, previous research has demonstrated the versatility of this tool. Therefore, it has been possible to use it in, for example, efficiency assessment of MH services [31], causal modelling of MH services [32] and application of the international comparison tool ESMS/DESDE (European Service Mapping Schedule/Description and Evaluation of Services and Directories) for assessing health care services and their impact on decision-making [33].

Online documents located on websites have a dynamic structure: some appear, some disappear, some are more or less strongly modified and so on. This target of study implies two analysis types: 1) compare the structural variations of the documents that still remain active in the corresponding, same, web sites, and 2) include new documents that match the designed search strategy. Considering the special nature of these documents and trying to make a robust analysis, the proposed adaptation of a standard systematic review can be an appropriate methodology rather than a scoping or narrative review [34–36].

The search strategy was designed following the fields established by the PICOS research question (population, intervention, comparator, outcomes and setting). The population (P) was MH services and systems (“mental health service*” OR “mental health system*”). According to this, only the online documents located in MH services and/or systems (governmental or institutions-related) have been included into the analysis. The intervention (I) was any international online MH strategy or recommendation designed to address the COVID-19 mental health impact (“strateg*” OR “recommendation*” AND “COVID-19” OR “COVID19” OR “COVID 19” OR “2019-nCOV” OR “SARS-COV-2”). The comparator (C) was not applicable in this review. The outcomes (O) refer to any online international report, document or guide (hereafter referred to as documents) that included global MH topics (“report*” OR “document*” OR “guideline*”). Last, the setting (S) comprises the countries included in the review: Australia, Canada, China, England, Finland, Greece, Ireland, Italy, Mexico, New Zealand, Portugal, Spain, Scotland and the United States (countries included in [37]). This fact was considered to make the results from both studies comparable and to establish a robust basement to start a potential benchmarking process.

In this review, for the vaccination period (second transversal cut of our analysis) and for comparison purposes, we applied the same search strategy developed in Almeda et al. 2021 [37], which consisted of “mental health service*” OR “mental health system” AND “strateg*” OR “recommendation* AND “COVID-19” AND “report” OR “document” OR “guideline”.

The search strategy was implemented on June 9, 2021, in Google. Two authors (NA and DDM) independently performed the search from different computers and cities to control for variability. Google© has been selected because it is the most popular platform to access to the content published on internet where governments, associations, NGOs, and institutions publish their online documents, including strategies and recommendations for addressing the impact of COVID-19 on MH. Additionally, the positive discrimination power of the method is assessed.

The inclusion criteria were online documents on general MH published by governments and international institutions, NGOs and associations developed to address the MH impact of the pandemic. Regarding language, this research only included guides published in English, Spanish, French, Italian and Portuguese because they are the original languages in the previous study [37]. The research team members have a complete knowledge of them. In addition to the inclusion criteria, online documents must be accessible to a general and/or specific target population. In the proposed methodology, the limit to define the accessibility of a specific potentially-selected document is established to the first 12 result pages offered by the Google© search engine. If the document is located beyond, it means that in practice the document does not exist because the target population rarely would access it.

To assess the evolution of the selected documents during the pandemic, all the documents included in the previous review (at a very early stage of the pandemic, first transversal cut) were re-evaluated. The period between the initial study (15 April 2020) and the current review (9 June 2021) was 16 months.

The exclusion criteria were documents that did not focus on general MH and did not provide online MH care delivery, strategies, recommendations or guidelines focused on specific areas such as economy and health workers.

As stated in previous research [37], the search strategy and eligibility criteria were checked and validated by experts in MH planning (psychologists, psychiatrists, senior managers and policy-makers) from the I-CIRCLE group (International CIty and urban Regional CoLlaborativE) and the PSICOST research group.

The quality of the selected documents has been assessed by answering two key questions: 1) have the strategy/ies and/or recommendation/s shown in the selected document well-defined and specific population target/s and coherent topics related to them? and 2) does it cover properly every item included in the checklist? If in both cases the document showed positive answers it has a good quality.

#### Document selection

NA and DDM conducted the selection process in the eligibility phase, reading the identification links and documents or full-text web pages. Any disagreement among reviewers was resolved by MRGC.

### Instrument

The questionnaire developed by Almeda, García-Alonso and Salvador-Carulla [37] was used to assess the structure and content of the selected documents. This instrument was created based on guidelines from the WHO, APA, UN, Centres for Disease Control and Prevention and MH Europe. It is composed of two checklists, one for symptoms and one for mental disorders, and a questionnaire of 39 items that can be organised into three main domains: 1) general COVID-19 information, 2) MH strategies and 3) MH recommendations. Domains 2 and 3 were both divided into two subdomains: i) MH topics, e.g., psychological health and anxiety, and ii) MH-related topics, e.g., issues related to people with disabilities and health care workers.

### Variable grouping

The items were categorised into seven indicator groups (IG) or variable sets. 1) Mental symptoms (IG1), 2) Mental disorders (IG2), 3) COVID-19 information (IG3), 4) MH strategies and MH topics (IG4), 5) MH strategies and MH-related topics (IG5), 6) MH recommendations and MH topics (IG6), and 7) MH recommendations and MH-related topics (IG7). A detailed description of the indicators and their categorisation can be found in the Supplementary Material.

### Data collection procedure

NA and DDM independently extracted data from the documents included in the review. The extracted values were mostly binary, where 1 meant “Yes” (when the information related to the item was included in the document) and 0 meant “No” (when the information related to the item was not included in the document). Discrepancies among NA and DDM were resolved by MRGC.

### Data analysis

To assess the evolution of the selected documents from April 2020 [37] (previously published) to June 2021 (from a very early stage of the pandemic to the vaccination period), a pre-post analysis was carried out using basic statistics and, for cases that fulfilled the normality assumption, Student’s t test for related samples.

A two-step cluster analysis was conducted to understand how documents were organised according to the seven IG. The distance used was log-likelihood, and the grouping method was the Akaike information criterion. Previously, Pearson’s chi-square test was used to determine the variable independence.

For all cases, the statistical significance level used was lower than 5% (*p* < 0.05). Statistical analyses were carried out using the software IBM SPSS Statistics, version 23.0 (IBM, Armonk, NY, USA) [38].

## Results

The results are structured into two sections according to the objectives of the study: 1) evolution of the documents and 2) systematic review and cluster analysis.

**Fig. 1:**
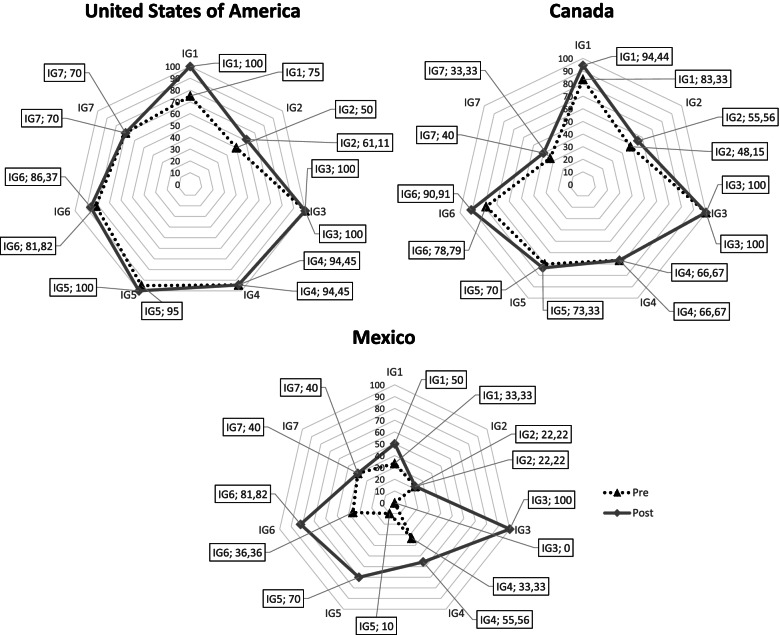
Longitudinal assessment for Canada, the United States of America and Mexico

1. Evolution of the documents during the pandemic (longitudinal analysis of the pre-existing documents)
  The previous review included 26 records, but 2 were removed because they were static documents. For that reason, 24 documents were re-evaluated in the present research.
  Statistically significant differences with a negligible effect size were found in the number of symptoms and mental disorders included in the documents (IG1 & IG2; t(23) = 3, *p* = 0.006, d = 0.18). We found an increase in these values in the vaccination stage (Mean = 8.75, SD = 3.25) with respect to those in the early stage of the pandemic (Mean = 8.21, SD = 2.9). A detailed analysis showed that the symptom with the greatest increase was bereavement, present in 9 to 15 documents (25%), followed by sleeping problems (from 14 to 16) and loneliness (from 17 to 18), while the rest of the symptoms remained stable. Regarding the analysed mental disorders, the presence of schizophrenia and psychotic disorders, bipolar disorders, chronic pain and obsessive–compulsive disorder increased in the selected documents up to 14.16%.
  Additionally, statistically significant differences with negligible effect size were found when the questions related to information, recommendations and strategies to cope with COVID-19 were analysed (IG3-IG7, t(23) = 2.24, *p* = 0.035, d = 0.19), with a higher number of questions included in the vaccination stage (Mean = 26.92, SD = 7.56) than in the early stage of the pandemic (Mean = 25.42, SD = 8.1). When each domain was studied separately, we found that every question related to “COVID-19 information” (IG3) increased its presence (100%), followed by the questions associated with “MH strategies and MH-related topics” (IG5), with an increase of up to 80% of that in the vaccination stage, highlighting the variable information for domestic violence victims with up to a 20.84% increase. Furthermore, 50% of the items included in “MH recommendations & MH topics” (IG6) increased in relevance, with provision of telephone or online contact with the general practitioner exhibiting the highest growth (25%). Here, Q17 was the only item that exhibited a reduced relevance because the provision of an online community forum was removed from one of the documents. Finally, the variables included in both “MH strategies and MH topics” (IG4) and “MH recommendations and MH-related topics” (IG7) increased up to 33% and 20%, respectively.
  In terms of the documents, most document subject matter remained constant throughout the pandemic (62.5%-95.83%, regarding the group of items) because they did not include new items, while 4.17%—33.33%, depending on the group of items, met the new criteria (new positive answers indicated that new items were included in the document). The number of symptoms (IG1) and variables linked to MH strategies and MH-related topics (IG5) and MH recommendations and MH topics (IG6) increased up to 33%; globally, institutions paid more attention to these topics. Only one document exhibited a reduced IG8 relevance by removing the online community forum.
  From an international point of view, Finland, Greece, Hong Kong, New Zealand, Portugal, and Switzerland did not improve their pre-existing documents. Ireland, England, Spain and Australia added small changes. Ireland included new positive answers in MH strategies and MH topics (IG4) and MH strategies and MH topics (IG6), with rates of 3.71% and 3.03%, respectively. England paid more attention to symptoms (IG1, 8.33%) and MH strategies and MH topics (IG4, 5.55%), but one variable from MH recommendations and MH topics (IG6) exhibited a reduced score (-4.54%). Spain added new symptoms (IG1, 5.55%), MH strategies and MH-related topics (IG5, 6.66%) and MH recommendations and MH topics (IG6, 3.03%). Finally, Australia added new symptoms (IG1, 5.55%), MH strategies and MH topics (IG4, 7.41%), MH strategies and MH-related topics (IG5, 13.33%) and MH recommendations and MH topics (IG6, 3.03%).
  The countries with the most important innovations (new positive answers mean that new items were included) in their pre-existing documents were Canada (from 68.61% to 74.42%), the United States of America (from 80.9% to 87.42%) and Mexico, showing the highest improvement from 25.03% to 59.94% (Fig. 1).
2. Systematic review and cluster analysis

### Document selection

**Fig. 2:**
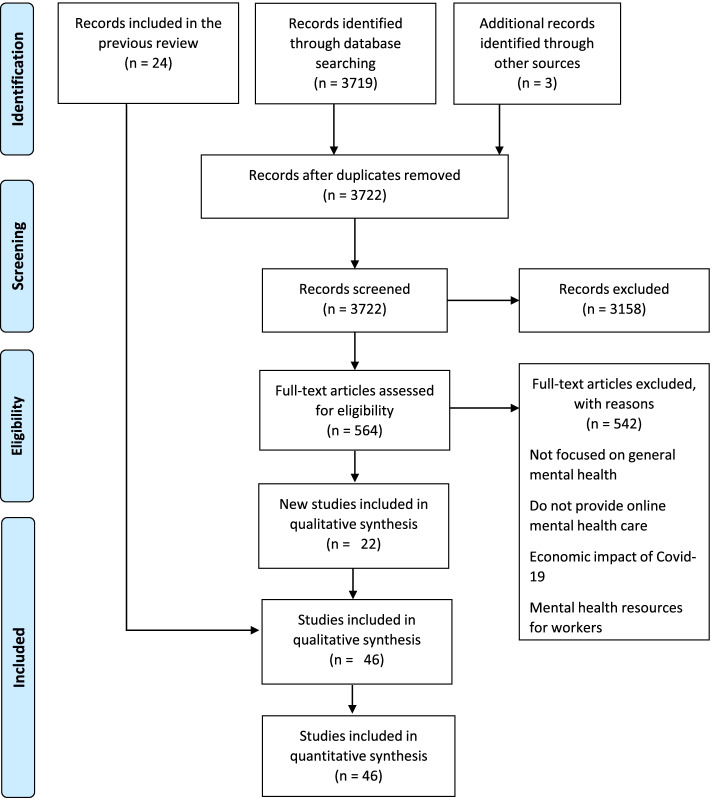
Flowchart and results. Adapted from Moher et al., 2009. Copyright 2009 by Moher et al.

A new search strategy was performed in Google© (June 9, 2021), and 3,722 records were identified. Additionally, three records were identified from the original documentation developed by international organizations such as WHO or UN. No duplicate records were found. In the eligibility phase, 22 new documents fulfilled the inclusion criteria. The new records and the updated documents from the previous review, resulting in a total of 46 documents, were included in the qualitative and quantitative analyses of the second transversal cut. By analysing these results, the positive discrimination power of the designed methodology has been successfully checked (Fig. 2).

### Document characteristics

Most of the 46 included documents had the “general population” as a target population (93.48%) and the “national level” as a territory target (93.48%) (see supplementary material, Table S1, for more details). The predominant format of the documents was a “web page” (86.96%). Documents 1, 6, 7, 27, 28, 31 and 34 were the most complete and, according to the quality criteria, are the best ones, while 8, 11, 38 and 44 shown the lower quality (see supplementary material, Table S2).

### Results of the cluster analysis (all documents in the second transversal cut)

The cohesion and separation profile were excellent (greater than 0.5) for each cluster analysis, and there were no outliers (Table 1). For more information on the distribution of each specific cluster (percentages of positive answers for each question), see the Supplemental Material (Tables S3, S4, S5, S6, S7, S8, and S9).

**Table 1:** Number of documents for each cluster and indicator group (IG)

Indicator group (IG)	Number of observations						
	Cluster 1	Cluster 2	Cluster 3	Cluster 4	Cluster 5	Cluster 6	Cluster 7
IG1 Mental symptoms	22	9	8	7			
IG2 Mental disorders	8	28	10				
IG3 Covid-19 information	43	3					
IG4 MH strategies and MH topics	9	8	8	8	7	6	
IG5 MH strategies and MH-related topics	10	11	9	16			
IG6 MH recommendations and MH topics	9	8	7	6	3	9	4
IG7 MH recommendations and MH-related topics	4	23	6	6	7		

### Mental symptoms (indicator Group 1, IG1)

In IG1, almost all documents included *stress* and *anxiety*, followed by *depression* (82.61%), *loneliness* (76.09%), *sleeping problems* (73.91%) and *bereavement* (63.04%).

Cluster 1 contains all broad-spectrum documents that include all symptoms, while Cluster 2 excludes *depression* and includes *bereavement* with a high proportion of negative answers, Cluster 3 includes sleeping problems and bereavement to a lesser extent *and* excludes *loneliness*, and Cluster 4 excludes *bereavement* (Table S3).

### Mental disorders (indicator Group 2, IG2)

For IG2 (mental disorders), the most common mental disorder in the selected documents was *anxiety disorder* (95.65%), followed by *depression* (78.26%) and *substance use* (67.39%). Regarding disorders present in less than half of the documents, *eating disorders* (47.83%) were followed by *schizophrenia*, *bipolar disorder* and *obsessive–compulsive disorders* (43.48% each). Finally, *chronic pain* and *dermatillomania* were the least relevant disorders (13.04% and 2.17%, respectively).

Cluster 1 focuses on broad-spectrum documents, and Cluster 2 excludes *chronic pain* and *dermatillomania*, while Cluster 3 represents the most specific strategies focused on *anxiety* and, to a lesser extent, *substance use disorder* and *eating disorder* (Table S4).

### COVID-19 information (indicator Group 3, IG3)

For IG3 (COVID-19 information), 85% of the documents (Cluster 1) included updated information on the COVID-19 situation and the government and global response, while the rest of the documents (Cluster 2) did not include it (Table S5).

### MH strategies & MH topics (indicator Group 4, IG4)

**Fig. 3:**
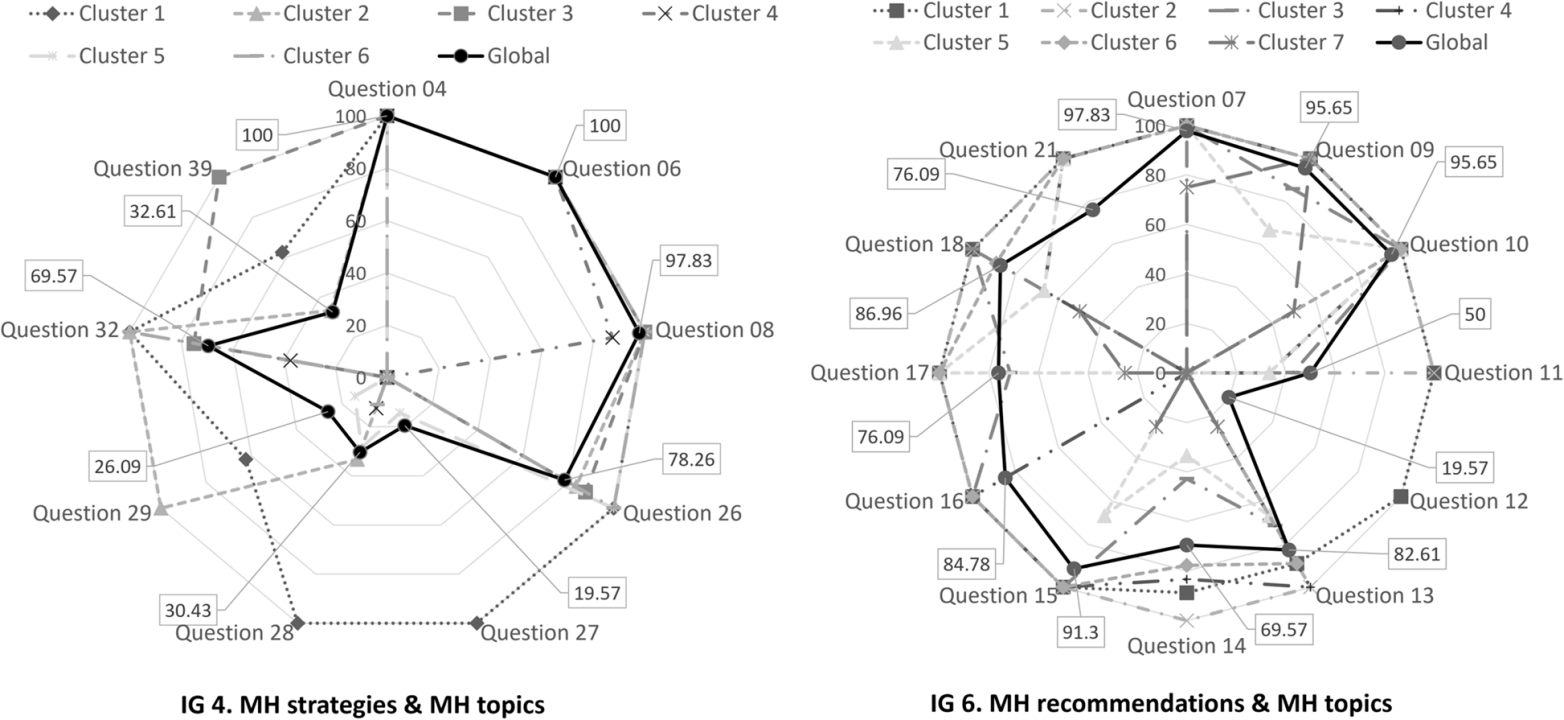
Percentages of positive answers for IG4 and IG6. For IG4 (MH strategies & MH topics) and globally speaking (dark black lines) questions Q4, Q6, Q8 and Q26 are very relevant for almost all the selected documents. For IG6 (MH recommendations & MH topics) documents are more complete including positive answers in all the questions except Q11 and Q12. There are specific differences when analysing different document groups (clusters)

For IG4 (MH strategies & MH topics), every selected document included positive answers for *tips for maintaining good MH* (Q4) and *described some psychological skills to help people cope with their anxiety and worry about COVID-19* (Q6)*,* and almost all *promoted social connection at home* (Q8, 97.83%) (global layer, Fig. 3). A second group of relevant strategies included positive answers for *information on how to support a loved one who is very anxious about COVID-19* (Q26) and *information on how to manage stress and anxiety* (Q32), with rates of 78.26% and 69.57%, respectively. Other important questions were Q39 (32.61%, *link for elderly people related to symptoms or mental disorders*) and Q28 (30.43%, *information on how to manage stress in case of positive test results*), while Q29 (26.09%, *how to reduce stigma*) and Q27 (19.57*%, stress management while people are waiting for COVID-19 test results*) were the least relevant.

Cluster 1 highlighted all the questions from IG4, while Cluster 2 excluded *stress management while people are waiting for COVID-19 test results* (Q27). Cluster 3 excluded Q27, *stress management strategies in the case of positive testing* (Q28) and *how to reduce stigma* (Q29) but highlighted the rest. Cluster 4 grouped the most specific documents, dominated by Q4, Q6 and Q8 but including Q28 and *information on how to manage stress and anxiety* (Q32), with a low proportion of positive answers (< 30%). Cluster 5 excluded Q32 and *links for elderly people related to symptoms or mental disorders* (Q39), emphasised *information on how to support a loved one who is very anxious about COVID-19* (Q26) and included, with a low proportion of positive answers, Q27, Q28 and Q29. Finally, Cluster 6 emphasised Q26 and Q32 but included Q28 with a low proportion (Table S6).

### MH strategies and MH-related topics (indicator Group 5, IG5)

IG5 showed a high representation of each of its items, including percentages greater than 50%. The most relevant questions were *information on how to maintain a healthy lifestyle* (Q5, 100%), *information for caregivers* (Q35, 82.61%) and *contemplated work at home* (Q38, 80.43%).

Clusters 1 and 2 included the most complete documents; however, Cluster 1 was focused on *information for health care workers* (Q30), *how to support health workers* (Q31) and *information for domestic violence victims* (Q34), while Cluster 2 highlighted all the questions in the IG. However, Cluster 3 excluded Q34, and Cluster 4 was the most specific, excluding Q31 and Q33 (*identifying health care staff needs*) but emphasising Q35 and Q38 (Table S7).

### MH recommendations and MH topics (indicator Group 6, IG6)

For IG6, most of the questions were highlighted (from 69.57% to 100%), except for Q11 (50%, *offer an online psychological assessment*) and Q12 (19.57%, *provide feedback on the psychological assessment results*) (Fig. 2).

Cluster 1 included broad-spectrum documents, while the rest of the clusters excluded Q12. Clusters 3, 4, 5 and 6 also excluded Q21 (*steps for understanding the child's feelings*), Q17 (*an online community forum*), Q16 (*telephone or online contact with other mental health professionals*) and Q11 (*offer an online psychological assessment)*, respectively. In contrast, Cluster 7 most specifically emphasised Q7 (*emotional support, such as conversations for sharing tips online*), Q9 and Q10 but included, with a high proportion of “NO” answers, Q13, Q15 and Q17 (Table S8).

### MH recommendations and MH-related topics (indicator Group 7, IG7)

The global profile of IG7 highlighted *information for parents* (Q19, 86.96%) and *how to explain the coronavirus to children* (Q20, 76.09%), followed by *alternatives to elder people to stay connected online* (Q22, 52.17%). Q23 (*help in getting established online and learning digital literacy skills*) and Q24 (*guidelines for COVID-19 outbreaks in residential care facilities*) were the least relevant for this IG (23.91% and 21.74%, respectively).

In IG7, Cluster 1 included broad-spectrum documents, while Cluster 3 excluded Q23 (*help in getting established online and learning digital literacy skills*) and Cluster 5 excluded Q24 (*guidelines for COVID-19 outbreaks in residential care facilities*). In contrast, Clusters 2 and 4 were the most specific. Cluster 2 emphasised *information for parents* (Q19), *how to explain the coronavirus to children* (Q20) and, to a lesser extent, *alternatives to elder people to stay connected online* (Q22), while Cluster 3 was focused on the last-mentioned question (Q22) (Table S9).

## Discussion

This research provides an overview of the evolution of international online documents that include MH strategies and recommendations to face the consequences of the COVID-19 pandemic on the population’s MH. Governments, international organisations and professional institutions have increased their interest in this topic over time. The increase in documents after the application of the search strategy in the vaccination stage (9^th^ June 2021) with respect to the baseline study (15 April 2020) is striking [37], growing from 88 records to 3,722 at the identification phase of the review.

The selected documents were mainly web pages and were focused on the general population, in accordance with the approach of a previous review [37]. However, the results showed a growing interest in specific topics (others have remained constant), but high structural heterogeneity and inequality were observed in the evolution of the pre-existing documents.

Focusing on documents evaluated in the longitudinal study, the evolution of symptoms points to greater attention to bereavement. As a result of the excess mortality resulting from the pandemic [1], governments and institutions began to articulate COVID-19 grief in their political contexts, including more information on bereavement symptoms. Sleeping problems were the second most common symptom and were more prevalent in the strategies and recommendations. The pandemic disrupted daily activities and caused sleeping problems as well as an increase in the use of sleeping drugs [39] and the overuse of media, bright blue light screens and television noise. During confinement, these behaviours have a negative impact on sleeping habits [40]. In addition, working from home or attending online classes also caused sleeping disturbances [41]. Additionally, some of the governments and institutions in charge of the selected documents found that loneliness was one of the most difficult and pervasive symptoms of COVID-19, which was partly derived from containment measures to control the spread of the virus [42]. Loneliness is also related to other MH symptomatology and has a negative impact on general wellbeing [43].

Regarding mental disorders, schizophrenia, bipolar disorder, chronic pain, and obsessive–compulsive disorder were the most frequently addressed in the documents from the early stage of the pandemic to the vaccination period. Governments and institutions have considered the inclusion of online support for people suffering from severe mental disorders.

Regarding the score changes in the instrument used [37], it is worth highlighting that information about the virus was increased in all the documents. The development of misleading and false information on COVID-19 has been significant [44]. It is crucial to have access to verified information to reduce the spread of fake news, which increases panic [45]. In particular, the WHO sent alerts regarding the spread of misleading information or an overabundance of information, making it more difficult to find trustworthy and verified information when needed [46].

However, Q14 (*Does it provide any telephone or online contact with the GP?*) was also more frequently included in the selected documents. This fact may be explained because, in gatekeeping systems, access to specialised MH care is through general practitioners, so it is a first step to access MH care provision. This indicates the great relevance of primary care services at the international level for the maintenance and continuity of care [47].

Last, the relevance of Q34 (*Does the strategy include information for domestic violence victims?*) also increased. During the pandemic, specifically during the lockdown, domestic violence increased because of the continuous contact among perpetrators and victims [48]. According to the United Nations Women, violence helplines and shelters worldwide reported an increase in requests for help [49], which was related to the results found in this review.

Large differences in strategy were found among countries. Mexico experienced the greatest increase in available online information, while that in most of the analysed countries remained constant. The main reason could be the temporary delay in the COVID-19 outbreak in the country [50] compared with that in Italy or England and that recommendations given by the WHO were not followed [51]. In addition, the lack of an established regulatory and legal framework to provide services such as telepsychiatry [51] could also delay the development of more integrative and comprehensive online MH documents (mainly guidelines). Although documents from England, Australia and the United States of America have not improved as much as those in Mexico, the initial version of these documents already included broader strategies that render them more difficult to improve. Additionally, England, Australia, the United States of America, and Canada developed a relevant number of new documents.

Regarding the vaccination period, the cluster analysis showed a distribution from broader to more specific documents in each IG. IG1 mainly included symptoms of stress and anxiety, which may be related to the high prevalence of these symptoms during the pandemic [52]. IG2 showed that anxiety, depression, and substance use disorders were the most frequent in the documents. In this case, containment measures related to COVID-19 and the negative impact on the population have resulted in the development of mental disorders as well as worsening of previous symptomatology [53]. Interestingly, IG3 demonstrates that most international governments and institutions included trustworthy information on COVID-19. This fact is crucial to fight against the spread of fake news not only because people often visit international health agencies and the Ministry of Health’s websites or read reports from them in search of credible information [54] but also because this could prevent conspiracy theories and misinformation that can complicate the vaccination process [55].

Moreover, in IG4, most of the documents included information on how to maintain good MH, how to cope with anxiety and worry about COVID-19 and how to promote social connection at home. More broader documents also addressed how to provide support to a loved one and how to reduce stigma related to COVID-19 infection, as well as provided information for elderly people. Scientific literature demonstrates that it is crucial to provide support to elderly people [56] as well as reduce the stigma associated with getting infected by COVID-19 [23]. Fortunately, some governments, such as England, Australia and Canada, are aware of this fact and include that information in their online documents. The most broader documents could be considered standards for developing and improving MH strategies and recommendations. IG5 integrated information on how to maintain a healthy lifestyle and work from home, as well as information for caregivers. These issues are relevant because the pandemic has had a negative, direct or indirect, impact on population wellbeing [57] through the modification of lifestyles, such as changes in eating habits and alcohol consumption [58], substance use [59] and body weight [60], which increase the number of risk factors for long-term health problems. These documents also respond to the need for information to cope with stressors and prevent burnout in informal [61] and formal caregivers [18, 62]. Additionally, the recommendation on working from home has changed the paradigm of many workers who have been pushed to virtually work or adopt a mixed model and, in many cases, increase their number of working hours, which can directly affect health [63].

In IG6, the most broader documents included information on offering an online psychological assessment and providing feedback on the results. The pandemic has pushed the adoption of an online MH care provision system to prevent the spread of the virus [6, 25, 64, 65]. MH services have changed or adapted their delivery of care. In this new context, the implementation of remote care is an urgent need [66]. To collaborate in reducing the spread of the virus during the pandemic, telepsychiatry was frequently implemented as an alternative for providing outpatient care [67]. A recent narrative review supports that the use of telepsychiatry could be an important tool to cope with the MH consequences of the pandemic [68].

Nevertheless, to provide online MH care, it is essential to offer an online assessment system as well as provide the results. Establishing and improving telemedicine services can have a major long-term positive influence on patient care that will persist after the pandemic [69]. However, the lack of clear policies and regulations regarding the delivery of tele-psychotherapy in many countries has been discouraging for therapists and potential users [70]. Few documents mention this resource, potentially because the telemedicine service has not yet been broadly implemented in these countries.

Finally, in IG7, most of the documents integrated information for parents, children and elderly individuals, with a group of documents focused exclusively on these topics. Greater attention to children and parents could be an answer from governments to the consequences of school closure [71] and social isolation and parental stress [72] to promote healthy development and prevent a stressful environment for children and their families [73]. Additionally, the information for elderly people is complemented by alternative options to stay connected to others through online platforms, which can have a positive effect and prevent the neurological and psychological impairment associated with the social isolation and loneliness from which this population group frequently suffers [74]. However, this strategy needs to be supported by learning digital literacy skills, presented in a lower percentage of the documents, to reduce the disparities among population groups in the use of the services provided by governments and institutions [75].

The results of this study should be interpreted considering some limitations.1) The selected documents analysed are all located on internet and cannot be found in any standard scientific database. In order to analyse them using rigorous fundamentals to make the results comparable, an adaptation of the standard systematic review methodology from Almeda et al. [37] was used. 2) The number of languages included in this analysis is another limitation due to translation problems. Russian-related and Asian languages were not included and further research is needed to provide a wider perspective. 3) Trying to make the results comparable, the countries selected for the second transversal cut (vaccination process) in the longitudinal analysis was established at the beginning of the vaccination period (Almeda et al. [37]). Obviously more countries developed online documents that can be also studied following the methodology. 4) The starting dates for vaccination were different among the selected countries, as well as the associated conditions and policies were not equally developed worldwide. For this study all the situations were similar enough to make it robust.

Further studies should focus on monitoring the online MH strategies and recommendations developed by governments and institutions during COVID-19 to assess the effect of the vaccination on international policies and guidelines. In addition, further studies should provide relevant information on the adequacy of the modifications and updates of these documents regarding the population needs of each country. Finally, this type of methodology should be a standard by which to determine not only the stability or growth of the published documents or the presence of new documents but also whether the initial governments’ positions and attitudes persist or change in relation to specific topics regarding economic, health or political factors (e.g., telehealth, telework, health worker support).

## Conclusions

To the best of our knowledge, this is the first study to collect empirical data from online international MH documents, guidelines and reports in a web page format, to cope with the MH impact of COVID-19 consequences from the early stage of the pandemic to the vaccination period and assess their evolution from 15 April 2020 to 9 June 2021.

The methodology (adaptation of the PRISMA guidelines) showed that these documents could be considered dynamic because they are subject to quick changes and modifications over time. The instrument applied for assessing documents, developed in a previous study [37], is sensitive enough to identify these changes (longitudinal analysis).

The evolution of the pandemic has been a challenge but also an opportunity for the different nations and global agencies to reassess how their resources are distributed and how society organises itself to recover from this crisis. To reach this goal, it is necessary to ensure the diversity of the investments and actions, such as by the development of more comprehensive guidelines and holistic MH programs, to find a balance among the interaction of MH, physical health, and social context that facilitate better MH in the population. For this aim, it is necessary to look beyond the biomedical paradigm and address the social determinants and individual necessities for people who suffer from or are at risk for a MH disorder.

## Supplementary Information

**Additional file 1:** **Table S1.** Documents included in the review. **Table S2**. Number of positive (YES) answers in percentage (%) per document and indicator group (IG). **TableS3**. Mental symptoms (indicator group 1, IG1). **TableS4**. Mental disorders (indicator group 2, IG2). **TableS5.** COVID-19 information (indicator group 3, IG3). **TableS6.** MH strategies & MH topics (indicator group 4, IG4). **TableS7**. MH strategies & MH-related topics (indicator group 5, IG5). **TableS8**. MH recommendations & MH topics (indicator group 6, IG6). **TableS9.** MH recommendations & MH-related topics (indicator group 7, IG7).

## Data Availability

All data supporting our findings will be shared on request. The documents included in the systematic review are available in the references section of the Supplementary Material.

## Abbreviations

MH: Mental health
WHO: World Health Organisation
APA: American Psychological Association
UN: United Nations
IG: Indicator group
