# Supplementary material for: A systematic review of the international evolution of online mental health strategies and recommendations during the COVID-19 pandemic
Source: BMC Psychiatry. 2022 Sep 20;22:621. doi: 10.1186/s12888-022-04257-8 (PMC9486794; doi:10.1186/s12888-022-04257-8)
Supplement: Supplementary file 1 — Additional file 1: Table S1. Documents included in the review. Table S2. Number of positive (YES) answers in percentage (%) per document and indicator group (IG). TableS3. Mental symptoms (indicator group 1, IG1). TableS4. Mental disorders (indicator group 2, IG2). TableS5. COVID-19 information (indicator group 3, IG3). TableS6. MH strategies & MH topics (indicator group 4, IG4). TableS7. MH strategies & MH-related topics (indicator group 5, IG5). TableS8. MH recommendations & MH topics (indicator group 6, IG6). TableS9. MH recommendations & MH-related topics (indicator group 7, IG7). [file 12888_2022_4257_MOESM1_ESM.pdf]

Supplementary material

Items included in the questionnaires:

IG1. Symptoms

- Loneliness
- Sleeping problems
- Anxiety
- Stress
- Bereavement
- Depression

IG2. Mental disorders

- Schizophrenia and psychotic disorders
- Anxiety disorder
- Bipolar disorders
- Depression
- Substance use disorder
- Chronic pain
- Eating Disorders
- Dermatillomania
- Obsessive compulsive disorder

IG3. COVID-19 general information

- Q1: Does the strategy include information related to the latest information on Covid-19?
- Q2: Does it include information on the strategies developed by the Government in response to the pandemic…)?
- Q3: Does it includes information on the latest information about the global response to the COVID-19 outbreak?

IG4. MH strategies and MH topics

- Q4: Include psychological tips for maintaining good mental health and coping with Covid-19?
- Q6: Describe some psychological skills to help people cope with anxiety and worry about COVID-19?

- Q8: Does it promote the social connection at home?
- Q26: Does the strategy include information on how to support a loved one who is very anxious about the COVID-19?
- Q27: Does the strategy include information on how to manage the stress while people await the test results?
- Q28: Does the strategy include information on how to manage the stress in case they have tested positive?
- Q29: Does the strategy include information on the stigma and how to reduce it?
- Q32: Does the strategy include information on how to manage stress and anxiety?
- Q39: Is there any link for elderly people related to any symptoms or mental disorders?

IG5. MH strategies and MH-related topics

- Q5: How to maintain a healthy lifestyle?
- Q25: Does it include special mention to disabled people?
- Q30: Does the strategy include information for healthcare workers?
- Q31: Does the strategy include information on how to support health care workers?
- Q33: Does the strategy develop any strategy for identifying healthcare staff needs as a result of the Coronavirus pandemic?
- Q34: Does the strategy include information for domestic violence victims?
- Q35: Does the strategy include information for caregivers?
- Q36: Does the strategy include any information on financial support for bussines/people affected by COVID-19?
- Q37: Does the strategy develop any advice on medication access during the Covid-19?
- Q38: Does the strategy contemplate work at home?

IG6: MH recommendations and MH topics

- Q7: Does it provide emotional support, such as conversations for sharing tips online?
- Q9: Describe how to access mental health services?
- Q10: Provide any phone or online mental health services?
- Q11: Does it offer an online psychological assessment?
- Q12: Does it provide feedback on the psychological assessment results?
- Q13: Does it provide any mental health treatment/intervention alternative?
- Q14: Does it provide any telephone or online contact with the general practitioner?

- Q15: Does it provide any telephone or online contact with the psychologist?
- Q16: Does it provide any telephone or online contact with other mental health professional?
- Q17: Does it provide an online community forum?
- Q18: Does it provide suicide and crisis support?
- Q21: Does it provide steps for understanding child's feelings?

IG7: MH recommendations and MH-related topics

- Q19: Does it include information for parents?
- Q20: Information on how to explain the coronavirus to children?
- Q22: Does it provide any alternative to elder people to stay connected online?
- Q23: Does it help in getting established online and learning digital literacy skills?
- Q24: Does it include any guideline for COVID-19 Outbreaks in residential care facilities (for people with physical and mental disabilities, other community based health facilities (e.g. Drug and alcohol services, community mental health)?

Documents characteristics

Table S1. Documents included in the review

Code	Authors (year)	Country[1]	Target Population[2]	Document Type[3]
**1**	Australian Government (1)	1	1	2
**2**	Centre for Addiction and Mental Health (2)	2	1	2
**3**	Ireland's Health Services (3)	8	1	2
**4**	Mental Health Ireland (4)	8	1	2
**5**	Gobierno de México (5)	10	1	2
**6**	Mental Health America (6)	16	1	2
**7**	Centers for Disease Control and Prevention (7)	16	1	2
**8**	MindHK (8)	3	1	1
**9**	Centre for Health Protection (9)	3	1	2
**10**	New Zealand Government (10)	11	1	2
**11**	Spanish Society of Psychiatry (11)	14	1	1
**12**	Psychology General Council of Spain (12)	14	1	2
**13**	Gov.UK (United Kingdom Government) (13)	4	1	2
**14**	Mental Health Foundation (14)	4	1	2
**15**	Government of Canada (15)	2	1	2
**16**	Nidirect Government Services (16)	8	1	2
**17**	Confédération suisse (17)	15	1	2
**18**	Psychografimata (18)	7	1	2
**19**	Official College of Psychology of Catalonia (19)	14	1	1
**20**	MIELI ry (20)	5	1	2
**21**	Ordem dos psicologos (21)	13	2	1
**22**	Beyond Blue (22)	1	1	2
**23**	Australian Psychological Society (23)	1	1	1
**24**	Mental Health Commission of Canada (24)	2	1	2
25	British Columbia (25)	2	1	2
26	Canadian Mental Health Association (26)	2	1	2
27	City of Toronto (27)	2	1	
28	Here to Help (28)	2	1	2
29	Center of innovation in Campus Mental Health (29)	2	1	2
30	U.S. Department of Health and Human Services (30)	16	1	2
31	Public Health Emergency (31)	16	1	2
32	Substance Abuse and Mental Health Services Administration (32)	16	1	2
33	Department of Health (33)	16	1	2
34	American Medical Association (34)	16	1	2
35	National Alliance of Mental Illness (35)	16	1	2
36	Life in Mind (36)	1	1	2
37	Phoenix Australia (37)	1	1	2
38	Lifeline (38)	1	1	2
39	Headspace (39)	1	1	2
40	Mental Health Coalition South Australia (40)	1	1	2
41	Better Health – every mind matters (41)	4	1	2
42	Centre for Mental Health (42)	4	1	2
43	Mayor of London – London Assembly (43)	4	1	2
44	Study UK (44)	4	1	2
45	Rethink Mental Illness (45)	4	1	2

46	Start a Conversation (46)	4	1	2

[1] Countries: (1) Australia, (2) Canada, (3) China: Hong Kong, (4) England, (5) Finland, (6) Germany, Switzerland and France; CORAASP and CLASS, (7) Greece, (8) Ireland, (9) Italy, (10) Mexico, (11) New Zealand, (12) Portugal, (13) Scotland, (14) Spain, (15) Switzerland and (16) United States of America.

[2] Target population: (1) General population and (2) Older adults.

[3] Document type: (1) Report and (2) Web page.

*In bold those documents included in the previous review.

Table S2. Number of positive (YES) answers in percentage (%) per document and indicator group (IG)

**Code**	**IG1**	**IG2**	**IG3**	**IG4**	**IG5**	**IG6**	**IG7**	**Total**
1	66.67	88.89	**100**	77.78	90	**100**	**100**	**89.05**
2	**100**	77.78	**100**	88.89	80	**100**	40	83.81
3	**100**	77.78	**100**	44.44	60	83.33	60	75.08
4	**100**	66.67	**100**	88.89	40	83.33	60	76.98
5	50	22.22	**100**	55.56	70	75	40	58.97
6	**100**	77.78	**100**	88.89	**100**	91.67	60	**88.33**
7	**100**	44.44	**100**	**100**	**100**	83.33	80	**86.82**
8	**100**	11.11	66.67	33.33	20	50	0	40.16
9	50	22.22	**100**	33.33	20	83.33	60	52.7
10	50	11.11	**100**	66.67	90	**100**	80	71.11
11	50	33.33	0	33.33	40	16.67	0	24.76
12	**100**	22.22	0	66.67	60	66.67	40	50.79
13	**100**	44.44	**100**	**100**	90	**100**	60	84.92
14	**100**	77.78	**100**	77.78	70	75	60	80.08
15	83.33	22.22	**100**	55.56	70	83.33	20	62.06
16	83.33	22.22	**100**	44.44	80	91.67	80	71.67
17	66.67	66.67	**100**	44.44	70	66.67	40	64.92
18	33.33	11.11	66.67	55.56	60	50	40	45.24
19	66.67	11.11	**100**	66.67	60	66.67	40	58.73
20	50	66.67	**100**	55.56	60	75	80	69.6
21	66.67	22.22	**100**	33.33	20	41.67	20	43.41
22	**100**	22.22	**100**	**100**	**100**	91.67	80	84.84
23	83.33	77.78	**100**	55.56	80	66.67	**100**	80.48
24	**100**	66.67	**100**	55.56	70	91.67	60	77.7
25	50	33.33	**100**	66.67	80	91.67	40	65.95
26	**100**	77.78	**100**	55.56	90	83.33	80	83.81
27	**100**	55.56	**100**	66.67	**100**	**100**	80	**86.03**
28	**100**	77.78	**100**	66.67	**100**	**100**	60	**86.35**
29	**100**	77.78	**100**	55.56	50	83.33	60	75.24
30	**100**	88.89	66.67	66.67	70	83.33	80	79.37
31	**100**	88.89	**100**	77.78	80	91.67	**100**	**91.19**
32	83.33	44.44	66.67	55.56	60	75	20	57.86
33	83.33	44.44	**100**	66.67	80	91.67	**100**	80.87
34	83.33	77.78	**100**	88.89	**100**	91.67	80	**88.81**
35	**100**	66.67	**100**	66.67	**100**	91.67	60	83.57
36	66.67	22.22	**100**	44.44	40	91.67	60	60.71
37	**100**	33.33	**100**	55.56	50	58.33	60	65.32
38	83.33	33.33	0	44.44	50	50	20	40.16
39	83.33	77.78	**100**	44.44	50	50	20	60.79
40	50	11.11	**100**	44.44	50	66.67	0	46.03
41	**100**	22.22	**100**	44.44	40	75	40	60.24
42	83.33	66.67	**100**	88.89	**100**	75	60	81.98
43	83.33	22.22	**100**	55.56	80	75	40	65.16
44	83.33	11.11	**100**	33.33	40	8.33	0	39.44

45	66.67	66.67	66.67	66.67	50	83.33	0	57.14
46	83.33	55.56	**100**	55.56	20	**100**	40	64.92

*In bold the highest scores per indicator group

Results of the cluster analysis

Table S3. Mental symptoms (indicator group 1, IG1)

Clusters	Loneliness	Sleeping problems	Anxiety	Stress	Bereavement	Depression
Cluster 1	100	95.45	95.45	100	100	100
Cluster 2	75	62.5	100	100	37.5	0
Cluster 3	0	33.33	100	100	44.44	100
Cluster 4	100	71.43	100	100	0	100
Global	76.09	73.91	97.83	100	63.04	82.61

*Percentage of documents that includes the question (positive answer for this specific question).

Table S4. Mental disorders (indicator group 2, IG2)

Clusters	Schizophrenia and psychotic disorders	Anxiety disorder	Bipolar disorders	Depression	Substance use disorder	Chronic pain	Eating Disorders	Dermatillomania	Obsessive-compulsive disorder
Cluster 1	37.5	75	50	100	100	75	50	12.5	50
Cluster 2	60.71	100	57.14	100	71.43	0	60.71	0	57.14
Cluster 3	0	100	0	0	30	0	10	0	0
Global	43.48	95.65	43.48	78.26	67.39	13.04	47.83	2.17	43.48

*Percentage of documents that includes the question (positive answer for this specific question).

Table S5. COVID-19 information (indicator group 3, IG3)

Clusters	Question 01	Question 02	Question 03
Cluster 1	100	95.35	93.02
Cluster 2	0	0	0
Global	93.48	95.35	93.02

*Percentage of documents that includes the question (positive answer for this specific question).

Table S6. MH strategies & MH topics (indicator group 4, IG4)

Clusters	Question 04	Question 06	Question 08	Question 26	Question 27	Question 28	Question 29	Question 32	Question 39
Cluster 1	100	100	100	100	100	100	62.5	100	62.5
Cluster 2	100	100	100	83.33	0	33.33	100	100	33.33
Cluster 3	100	100	100	87.5	0	0	0	75	100
Cluster 4	100	100	87.5	0	0	12.5	0	37.5	0
Cluster 5	100	100	100	100	14.29	28.57	14.29	0	0
Cluster 6	100	100	100	100	0	11.11	0	100	0
Global	100	100	97.83	78.26	19.57	30.43	26.09	69.57	32.61

*Percentage of documents that includes the question (positive answer for this specific question).

Table S7. MH strategies & MH-related topics (indicator group 5, IG5)

Clusters	Question 05	Question 25	Question 30	Question 31	Question 33	Question 34	Question 35	Question 36	Question 37	Question 38
Cluster 1	100	30	90	90	60	80	50	40	20	40
Cluster 2	100	81.82	100	100	90.91	100	100	90.91	100	100
Cluster 3	100	77.78	100	100	77.78	0	100	66.67	55.56	100
Cluster 4	100	25	12.5	0	0	31.25	81.25	68.75	50	81.25
Global	100	50	67.39	63.04	50	52.17	82.61	67.39	56.52	80.43

*Percentage of documents that includes the question (positive answer for this specific question).

Table S8. MH recommendations & MH topics (indicator group 6, IG6)

Cluster	Question 07	Question 09	Question 10	Question 11	Question 12	Question 13	Question 14	Question 15	Question 16	Question 17	Question 18	Question 21
Cluster 1	100	100	100	100	100	88.89	88.89	100	100	100	100	100
Cluster 2	100	100	100	100	0	100	100	100	100	100	100	100
Cluster 3	100	85.71	100	42.86	0	71.43	42.86	100	100	71.43	100	0
Cluster 4	100	100	100	33.33	0	100	83.33	100	100	0	66.67	100
Cluster 5	100	66.67	100	33.33	0	66.67	33.33	66.67	0	100	66.67	100
Cluster 6	100	100	100	0	0	88.89	77.78	100	100	100	88.89	100
Cluster 7	75	100	50	0	0	25	0	25	0	25	50	0
Global	97.83	95.65	95.65	50	19.57	82.61	69.57	91.3	84.78	76.09	86.96	76.09

*Percentage of documents that includes the question (positive answer for this specific question).

Table S9. MH recommendations & MH-related topics (indicator group 7, IG7)

Clusters	Question 19	Question 20	Question 22	Question 23	Question 24
Cluster 1	100	100	100	100	100
Cluster 2	100	78.26	43.48	0	0
Cluster 3	100	100	83.33	0	100
Cluster 4	0	0	16.67	0	0
Cluster 5	100	100	57.14	100	0
Global	86.96	76.09	52.17	23.91	21.74

*Percentage of documents that includes the question (positive answer for this specific question).

References extracted from the systematic review

1. Australian Government. Head to Health COVID-19 Support [Internet]. 2020. Available from: https://headtohealth.gov.au/Covid-19-support/Covid-19 %0D%0A

2. Centre for Addiction and Mental Health. Mental Health and the COVID-19 Pandemic [Internet]. 2020. Available from: https://www.camh.ca/en/health-info/mental-health-and-covid-19

3. Ireland's Health Services. Your mental health. Advice on how to mind your mental health during the coronavirus outbreak [Internet]. 2020. Available from: https://www2.hse.ie/mental-health/

4. Mental Health Ireland. Minding Our Mental Health during COVID-19 [Internet]. 2020. Available from: https://www.mentalhealthireland.ie/get-support/covid19/

5. Gobierno de México. Salud Mental [Internet]. 2020. Available from: https://coronavirus.gob.mx/salud-mental/

6. Mental Health America. Mental Health And COVID-19-Information And Resoruces [Internet]. 2020. Available from: https://mhanational.org/covid19

7. Centers for Disease Control and Prevention. Coronavirus Disease 2019 (COVID-19) [Internet]. 2020. Available from: https://www.cdc.gov/coronavirus/2019-ncov/daily-life-coping/managing-stress-anxiety.html?CDC_AA_refVal=https%3A%2F%2Fwww.cdc.gov%2Fcoronavirus%2F2019-ncov%2Fprepare%2Fmanaging-stress-anxiety.html

8. MindHK. Managing our Mental Health & Staying Well during a Virus Outbreak. Responding to 2019-nCOV [Internet]. 2020. Available from: https://www.mind.org.hk/wp-

content/uploads/2020/02/ResponseCoronavirusENG6.2.2020-.pdf

9. Centre for Health Protection. Mental Health Infostation [Internet]. 2020. Available from: https://www.chp.gov.hk/mhi/index_en.html

10. New Zealand Government. Ways we're uniting agains COVID-19 [Internet]. 2020. Available from: https://covid19.govt.nz/

11. Spanish Society of Psychiatry. CUIDE SU SALUD MENTAL DURANTE LA CUARENTENA POR CORONAVIRUS [Internet]. 2020. Available from: http://www.sepsiq.org/file/InformacionSM/SEP COVID19-Salud Mental Cuarentena.pdf %0D%0A

12. Psychology General Council of Spain. Orientaciones para la gestión psicológica de la cuarentena por el Coronavirus [Internet]. 2020. Available from: http://www.infocop.es/view_article.asp?id=8655

13. GOV.UK. Guidance for the public on the mental health and wellbeing aspects of coronavirus (COVID-19) [Internet]. 2020. Available from: https://www.gov.uk/government/publications/covid-19-guidance-for-the-public-on-mental-health-and-wellbeing/guidance-for-the-public-on-the-mental-health-and-wellbeing-aspects-of-coronavirus-covid-19

14. Mental Health Foundation. How to look after your mental health during the Coronavirus outbreak [Internet]. 2020. Available from: https://www.mentalhealth.org.uk/coronavirus

15. Government of Canada. Taking care of your mental health during COVID-19 [Internet]. 2020. Available from: https://www.canada.ca/en/public-health/services/publications/diseases-conditions/taking-care-mental-health.html

16. Nidirect Government Services. Coronavirus (COVID-19): taking care of your mental health and wellbeing [Internet]. 2020. Available from: https://www.nidirect.gov.uk/articles/coronavirus-Covid-19-taking-care-your-mental-health-and-wellbeing

17. Confédération suisse. Nouveau coronavirus: Recommandations pour la vie quotidienne [Internet]. 2020. Available from: https://www.bag.admin.ch/bag/fr/home/krankheiten/ausbrueche-epidemien-pandemien/aktuelle-ausbrueche-epidemien/novel-cov/empfehlungen-fuer-den-alltag.html

18. Psychografimata. Corona: 8 ways to take care of your mental health [Internet]. 2020. Available from: https://www.psychografimata.com/κορonaϊός-8-τρόποι-να-φροντίσετε-την-ψυ/

19. Official College of Psychology of Catalonia. Recomendaciones de gestión psicológica durante cuarentenas por enfermedades infecciosas [Internet]. 2020. Available from: https://www.copc.cat/adjuntos/adjunto_15214/v/Guía de gestión psicológica frente a cuarentenas por enfermedades infecciosas- Español.pdf?tm=1584361973

20. MIELY ry. Koronaviruksen ai-heuttama huolta voi lievittää [Internet]. 2020. Available from: https://mieli.fi/fi/koronaviruksen-aiheuttamaa-huolta-voi-lievittää

21. Ordem dos Psicologos. COVID-19 COMO LIDAR COM UMA SITUAÇÃO DE ISOLAMENTO SE FOR UM CIDADÃO SÉNIOR (OU SEU FAMILIAR) [Internet]. 2020. Available from: https://www.ordemdospsicologos.pt/ficheiros/documentos/covid_19_seniores.pdf

22. Beyond Blue. Coronavirus Mental Wellbeing Support Service [Internet]. 2020. Available from: https://coronavirus.beyondblue.org.au/

23. Australian Psychological Society. Coronavirus (COVID-19) information for Australians [Internet]. 2020. Available from: https://www.psychology.org.au/COVID-19-Australians

24. Mental Health Comission of Canada. Resource Hub: Mental health and wellness during the COVID-19 pandemic [Internet]. 2020. Available from: https://www.mentalhealthcommission.ca/English/covid19

25. Bristish Columbia. Virtual mental health supports [Internet]. 2021 [cited 2021 Sep 15]. Available from: https://www2.gov.bc.ca/gov/content/health/managing-your-health/mental-health-substance-use/virtual-mental-health-supports

26. Canadian Mental Health Association. Everyone deserves to feel well [Internet]. 2021 [cited 2021 Sep 15]. Available from: https://cmha.ca/

27. City of Toronto. COVID-19: Mental Health Resources [Internet]. 2021 [cited 2021 Sep 15]. Available from: https://www.toronto.ca/community-people/health-wellness-care/covid-19-wellness-during-the-pandemic/covid-19-mental-health-resources/

28. BC Partners for Mental Health and Substance Use Information. Here to help - Mental health and substance use information you can trust [Internet]. 2021 [cited 2021 Sep 15]. Available from: https://www.heretohelp.bc.ca/covid-19-mental-health-supports

29. Center for Innovation in Campus Mental Health. COVID-19 Resources [Internet]. 2021 [cited 2021 Sep 15]. Available from: https://campusmentalhealth.ca/covid-19-resources/

30. U.S. Department of Health & Human Services. COVID-19 VACCINES ARE SAFE, EFFECTIVE, AND FREE [Internet]. 2021 [cited 2021 Sep 15]. Available from: https://www.hhs.gov/

31. Public Health Emergency. Mental and Behavioural Health - 2019 Novel Coronavirus Resources [Internet]. 2021 [cited 2021 Sep 15]. Available from: https://www.phe.gov/emergency/events/COVID19/mental-behavioral-health/Pages/default.aspx

32. Substance Abuse and Mental Health Services Administration. Get help [Internet]. 2021 [cited 2021 Sep 15]. Available from: https://www.samhsa.gov/

33. Department of Health. Supporting Mental Well-being During COVID-19 [Internet]. 2021 [cited 2021 Sep 15]. Available from: https://www.health.state.mn.us/communities/mentalhealth/support.html

34. American Medical Association. Managing mental health during COVID-19 [Internet]. 2021 [cited 2021 Sep 15]. Available from: https://www.ama-assn.org/delivering-care/public-health/managing-mental-health-during-covid-19

35. National Alliance on Mental Illness. From Next Door To Across The Nation [Internet]. 2021 [cited 2021 Sep 15]. Available from: https://nami.org/Home

36. Life in Mind. Coronavirus (COVID-19) mental health support [Internet]. 2021 [cited 2021 Sep 15]. Available from: https://lifeinmind.org.au/communities/support-for-those-impacted-by-adverse-events/mental-health-support-for-covid-19

37. Phoenix Australia. Resources Coronavirus (COVID-19) [Internet]. 2021 [cited 2021 Sep 15]. Available from: https://www.phoenixaustralia.org/resources/coronavirus-covid-19/

38. Lifeline. Mental health and wellbeing during the Coronavirus COVID-19 outbreak [Internet]. 2021 [cited 2021 Sep 15]. Available from: https://www.lifeline.org.au/get-help/information-and-support/covid-19/

39. Headspace. How to cope with stress related to Coronavirus (COVID-19) [Internet]. 2021 [cited 2021 Sep 15]. Available from: https://headspace.org.au/young-people/how-to-cope-with-stress-related-to-covid-19/

40. Mental Health Coalition South Australia. COVID-19: Mental health in uncertain times [Internet]. 2021 [cited 2021 Sep 15]. Available from: https://mhcsa.org.au/covid-19-mental-health-in-uncertain-times/

41. Better Health - every mind matters. Mental wellbeing while staying at home [Internet]. 2021 [cited 2021 Sep 15]. Available from: https://www.nhs.uk/every-mind-matters/coronavirus/mental-wellbeing-while-staying-at-home/

42. Centre for Mental Health. Coronavirus: resources and information [Internet]. 2021 [cited 2021 Sep 15]. Available from: https://www.centreformentalhealth.org.uk/coronavirus-resources-and-information

43. Mayor of London - London Assembly. Coronavirus and looking after your mental health [Internet]. 2021 [cited 2021 Sep 15]. Available from: https://www.london.gov.uk/coronavirus/coronavirus-and-looking-after-your-mental-health

44. Study UK. Covid-19: Information for international students [Internet]. 2021 [cited 2021 Sep 15]. Available from: https://study-uk.britishcouncil.org/moving-uk/coronavirus

45. Rethink Mental Illness. Coronavirus and mental health [Internet]. 2021 [cited 2021 Sep 24]. Available from: https://www.rethink.org/advice-and-information/covid-19-support/

46. Start a Conversation. Mental Health Support During COVID-19 lockdown [Internet].

2021 [cited 2021 Sep 24]. Available from:

https://www.startaconversation.co.uk/coronavirus-and-mental-wellbeing
